# Supplementary material for: A Multi-Omics Approach Identifies Key Regulatory Pathways Induced by Long-Term Zinc Supplementation in Human Primary Retinal Pigment Epithelium
Source: Nutrients. 2020 Oct 6;12(10):3051. doi: 10.3390/nu12103051 (PMC7601425; doi:10.3390/nu12103051)
Supplement: Supplementary file 1 [file nutrients-12-03051-s001.pdf]

nutrients

Supplementary material

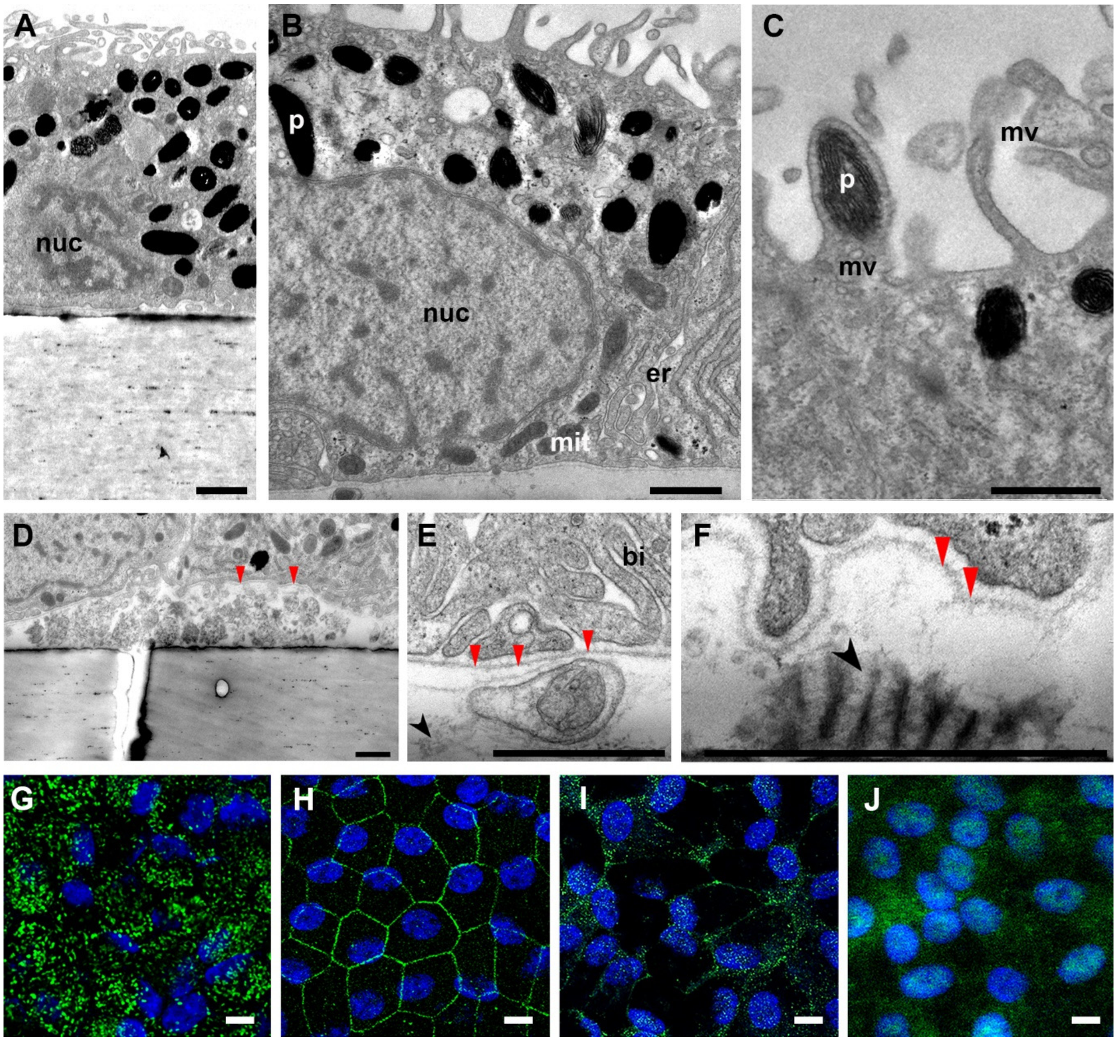

Supplementary Figure 1. Characterisation of the human primary RPE cell model. Cells were cultured on 10 um thick polyester membrane inserts with 0.4 um pores. Primary RPE developed microvilli (mv) (**A**), high pigmentation (p) (**B–C**), basal infoldings (bi) (**E**), basal lamina (red arrowheads), high metabolic activity (rough endoplasmic reticulum (ER), mitochondria (mit), vesicles (mv)) (**B–F**). RPE signature protein expression shown in green in *in vitro* RPE culture for pMEL-17 (**G**), ZO-1 (**H**), BEST-1 (**I**) and RPE65 (**J**). RPE flatmounts were counterstained with DAPI shown in blue (**G–J**). Dome-shaped deposit accumulation in sub-RPE space with condensed deposits (black arrowheads) banding material (black arrowhead) fibrillar deposits (black arrowhead) and different multicomponent or amorph vesicles (**D**). Black scale bar is 1 μm, white scalebar is 10 μm.

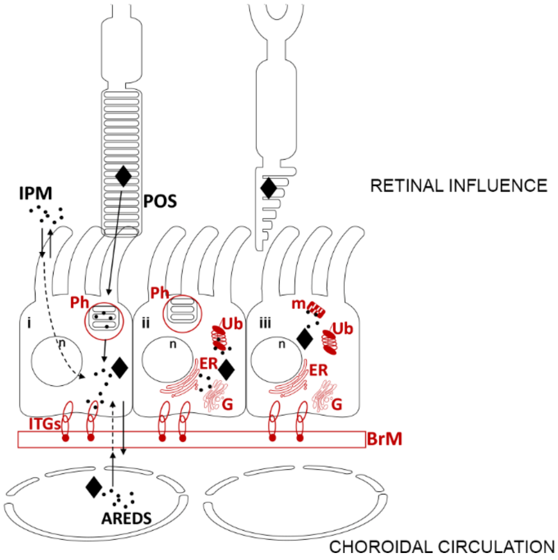

Supplementary Figure 2. Regulatory cellular biological processes. Red highlights the affected biological pathways upon long-term zinc supplementation, as phagosome maturation and cellular location/extracellular matrix organisation (**i**), protein processing/transport (**ii**), oxidative stress response (**iii**). POS, photoreceptor outer segments, IPM, interphotoreceptor matrix, BrM, Bruch's membrane, Ph, Phagosome, ER, Endoplasmic reticulum, G, Golgi, Ub, Ubiquitin-proteasome system, ITGs, Integrins, m, mitochondria, available Zn2+ (●), Zinc buffering/muffling (◆), AREDS, Age-Related Eye Disease Study.

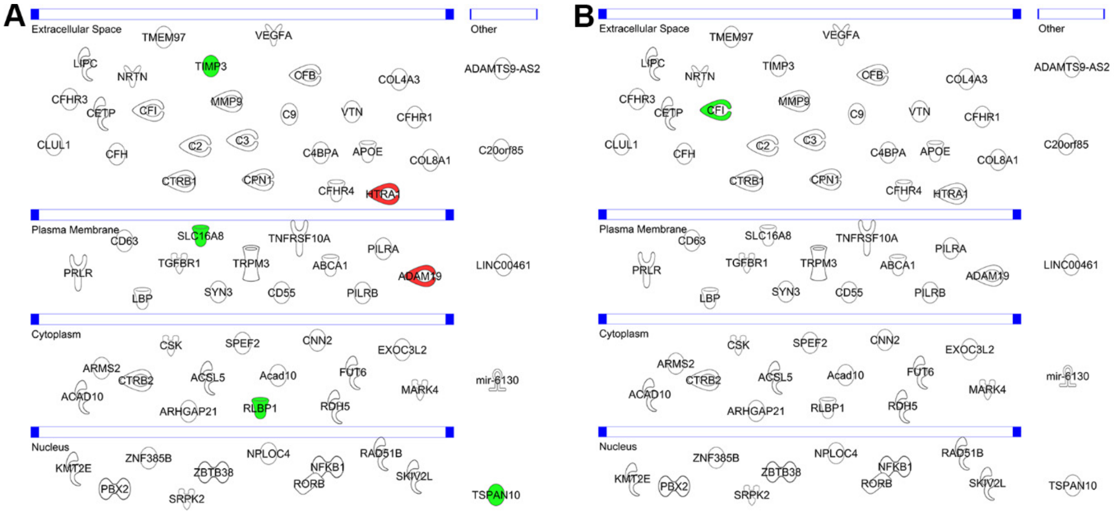

Supplementary Figure 3. Significant changes of AMD associated genes upon zinc apical **(a)** or basal **(b)** supplementation. Dataset of differentially expressed/secreted molecules were overlaida with the hits from recent GWAS studies on AMD [1–3].

References

1. Fritsche, L.G.; Igl, W.; Bailey, J.N.; Grassmann, F.; Sengupta, S.; Bragg-Gresham, J.L.; Burdon, K.P.; Hebbring, S.J.; Wen, C.; Gorski, M., et al. A large genome-wide association study of age-related macular degeneration highlights contributions of rare and common variants. *Nat Genet.* **2016**, *48*, 134–143.
2. Lorés-Motta, L.; Paun, C.C.; Corominas, J.; Pauper, M.; Geerlings, M.J.; Altay, L.; Schick, T.; Daha, M.R.; Fauser, S.; Hoyng, C.B., et al. Genome-Wide Association Study Reveals Variants in CFH and CFHR4

Associated with Systemic Complement Activation: Implications in Age-Related Macular Degeneration. *Ophthalmology*. **2018**, *125*, 1064–1074, doi:10.1016/j.ophtha.2017.12.023.

3. Han, X.; Gharahkhani, P.; Mitchell, P.; Liew, G.; Hewitt, A.W.; MacGregor, S. Genome-wide meta-analysis identifies novel loci associated with age-related macular degeneration. *J Hum Genet.* **2020**, *65*, 657–665, doi:10.1038/s10038-020-0750-x.

© 2020 by the authors. Submitted for possible open access publication under the terms and conditions of the Creative Commons Attribution (CC BY) license (http://creativecommons.org/licenses/by/4.0/).
